# What are the primary roles of peroxidases in enzymatic browning of fresh-cut fruits and vegetables, browning promoters or ROS scavengers?

**DOI:** 10.3389/fnut.2026.1859645

**Published:** 2026-06-12

**Authors:** Xiao Yuan, Zhaorui Zhu, Siju Wang, Xinjie Li, Jinsheng Cheng, Bin Wang

**Affiliations:** 1 College of Biology and Agriculture, Shaoguan University, Shaoguan, China; 2 School of Food Science, Shaoguan University, Shaoguan, China; 3 Guangdong Provincial Key Laboratory of Utilization and Conservation of Food and Medicinal Resources in Northern Region, Shaoguan University, Shaoguan, China; 4 Guangdong Provincial Engineering and Technology Research Center of Special Fruit and Vegetables in Northern Region, Shaoguan University, Shaoguan, China

**Keywords:** enzymatic browning, fresh-cut produce, peroxidase, phenolic oxidation, ROS metabolism

## 1 Introduction

Fresh-cut fruits and vegetables, which undergo processes such as washing, peeling, cutting or slicing, packaging, and subsequent cold storage, represent a dynamic and expanding segment of the fresh produce industry (1). Despite minimal processing, they retain their raw, fresh state, offering consumers a valuable combination of high nutritional value, convenience, and superior flavor (2). However, the physical disruption of tissue integrity during processing alters their physiological behavior and storage requirements, significantly shortening their shelf life compared to the intact produce (3). Consequently, maintaining and even enhancing the safety and quality of fresh-cut fruits and vegetables necessitates rigorous management of enzymatic activity, respiration, and microbial proliferation.

Among these quality challenges, enzymatic browning, characterized by undesirable dark discoloration, remains a primary concern (4). This adverse change diminishes both the visual appeal and nutritional quality of fresh-cut produce (5), leading to substantial postharvest losses. Fresh-cut processing inevitably compromises tissue integrity, allowing for direct contact between phenolic compounds and oxidases, particularly phenol oxidases, which are normally separated into distinct subcellular organelles through the compartmentalization of various membranes (6). Upon wounding, the release of phenolic compounds and phenolases triggers immediate oxidation in the presence of atmospheric oxygen within plant cells. Oxidative enzymes, particularly polyphenol oxidase (PPO) and certain peroxidases, catalyze the oxidation of such phenolic compounds into highly reactive quinones, which then polymerize to form brown melanin-like polymers or other dark pigments (7).

While it is well-established that phenolase-mediated reactions drive the browning process, research has historically prioritized the role of PPO (8–10). Given the multifunctional roles of peroxidase in plant systems, its specific contribution to enzymatic browning remains poorly understood, and its potential as a regulatory target has yet to be fully exploited. Therefore, this opinion aims to review the multifaceted roles of peroxidase

in enzymatic browning, highlighting its significance as a potential target for controlling browning in fresh-cut produce. Additionally, we propose that peroxidases act as context-dependent modulators, with their effect determined by the specific isoenzyme, subcellular localization, or substrate specificity.

## 2 Classification and multifaceted roles of peroxidase

### 2.1 Peroxidase constitutes a multienzyme family

**Figure 1:**
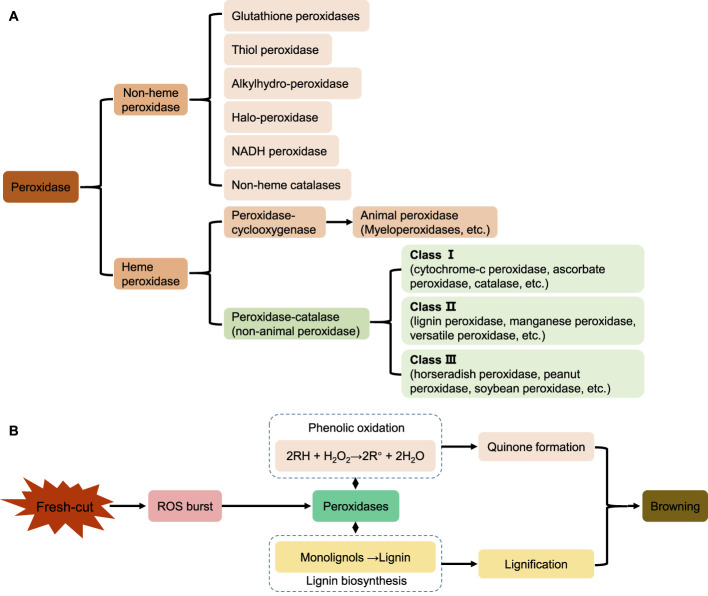
The classification **(A)** of peroxidase and its potential roles **(B)** in regulating browning. RH is a substrate for peroxidase, and R° is a free-radical product derived from it. The images were prepared based on the published works (11, 43).

Peroxidases are enzymes that decompose H_2_O_2_ while oxidizing a broad spectrum of phenolics and non-phenolic substrates like lignin and amine compounds. Peroxidases are ubiquitous, found in almost every living organism, from bacteria, fungi, animals, algae, and plants (11). Based on whether they contain a heme prosthetic group, peroxidases are classified as either heme or non-heme enzymes (12, 13). According to the RedoxiBase database, more than 80% of characterized peroxidase genes encode heme-containing proteins (14). Non-heme peroxidases, such as glutathione peroxidases (GPX), thiol peroxidase, alkylhydroperoxidase, halo-peroxidase, NADH peroxidase, and non-heme catalases (Figure 1A), constitute only a minor fraction (15–17).

Heme peroxidases are further divided into two subfamilies: the peroxidase-cyclooxygenase and the peroxidase-catalase (Figure 1A). Peroxidase-cyclooxygenases are found only in animals and are believed to play a role in host defense and innate immunity responses (18). This family mainly comprises four enzymes: myeloperoxidase, lactoperoxidase, thyroid peroxidase, and eosinophil peroxidase (11).

Peroxidase-catalases are a group of non-animal heme peroxidases that have attracted the most intensive study in past years. Originally named as “plant, fungal and bacterial heme peroxidases,” this family has since been renamed peroxidase-catalase (11). Members of this family are divided into three classes: class I, II, and III. Class I peroxidases, which include cytochrome-c peroxidase, ascorbate peroxidase (APX), and catalase (CAT), are intracellular enzymes that play a major role in detoxifying reactive oxygen species (ROS), particularly H_2_O_2_ (19– 21). Their unique catalytic capacity to dismute H_2_O_2_ and to generate molecular oxygen from H_2_O_2_ protects cells against oxidative stress.

Class II peroxidases are fungal extracellular enzymes that principally include lignin peroxidase, manganese peroxidase, and versatile peroxidase (Figure 1A). This class of peroxidases is central to the biodegradation and depolymerization of lignin in fungi (22). Manganese peroxidases, also secreted by these lignin-degrading fungi, catalyze the peroxide-dependent oxidation of Mn^2^^+^ to Mn3^+^ (23). Versatile peroxidases possess a hybrid molecular architecture that combines features of both lignin and manganese peroxidases. Unlike manganese peroxidases, which are specific for Mn^2^^+^ versatile peroxidases can oxidize phenolic and non-phenolic substrates, such as lignin, in the absence of manganese (24). Apparently, peroxidases in this class are not involved in browning reactions of fresh-cut produce.

Class III peroxidases are abundant in the plant kingdom and include enzymes such as soybean peroxidase, horseradish peroxidase, peanut peroxidase, and others (Figure 1A). Studies have shown that peroxidases of this class perform a broad array of functions related to plant growth, development, and stress defense (25). These roles encompass the metabolism of ROS and reactive nitrogen species (26), modulation of plant growth and stress responses (27, 28), lignification (29–31), wound healing (32), and more.

### 2.2 Multifaceted roles of peroxidase in enzymatic browning

Enzymatic browning of fresh-cut fruits and vegetables typically involves ROS metabolism, phenolic oxidation, and lignification, often occurring simultaneously or in combination (7, 9, 33). Given their broad catalytic range across the cell wall, vacuole, and apoplast, peroxidases may play important complementary roles in enzymatic browning, depending on species and storage conditions (34).

#### 2.2.1 Peroxidases-driven ROS metabolism and/or phenolic oxidation regulate enzymatic browning

ROS are highly oxidative molecules that can damage plant tissues, while cutting induces a ROS burst and accumulation in fresh fruits and vegetables (35). Elevated ROS levels can cause significant oxidative damage, particularly by compromising the integrity and functionality of cellular membranes. This breakdown facilitates direct interactions between phenolic substrates and oxidative enzymes, thereby catalyzing the enzymatic browning process (36). Consequently, managing ROS is crucial for reducing browning and maintaining the quality of fresh-cut fruits and vegetables (6).

Peroxidases play a critical role in maintaining ROS balance and protecting plants against oxidative stress. Certain peroxidases, particularly non-heme peroxidases like GPX and Class I heme peroxidases such as APX and CAT, solely function in ROS metabolism, notably H_2_O_2_, thereby regulating browning in fresh-cut produce. GPX and APX reduce H_2_O_2_ by directly oxidizing reduced glutathione and ascorbic acid, respectively, using them as co-substrates within plant cells (37). CAT uniquely utilizes H_2_O_2_ as its sole substrate, catalyzing its degradation to water and oxygen, a process often originating from peroxisomal oxidases (38, 39). Consistent with this, the direct application of CAT to fresh-cut potatoes has been shown to reduce browning by diminishing H_2_O_2_ level (40). These specific peroxidases scavenge ROS to maintain membrane integrity, thereby mitigating browning.

Beyond their role in ROS metabolism, specific peroxidases oxidize phenolic compounds using H_2_O_2_ as a co-substrate (41). For instance, horseradish peroxidase catalyzes the oxidation and polymerization of aqueous aromatic compounds in the presence of H_2_O_2_ (42), a process in which H_2_O_2_ levels decrease as phenolics are oxidized. This functional divergence, where peroxidases act as ROS scavengers that attenuate browning yet also as phenolic oxidizers that promote it, presents a central paradox that may lead to misinterpretations in the literature. We argue that these seemingly conflicting findings primarily may stem from the dynamic fluctuations in local substrate concentrations, particularly the ratio of H_2_O_2_ to phenolic compounds, which ultimately dictates whether peroxidases function as ROS scavengers or pro-oxidant catalysts.

#### 2.2.2 Peroxidase-mediated lignin biosynthesis and polymerization contribute to browning

Lignin accumulation and/or lignification are also proposed to be associated with plant tissue browning (Figure 1B) and class III peroxidases have essential roles in plant lignification (43). For instance, an increase in lignin biosynthesis has been reported to result in the formation of russet spots in pear fruits (44). Cutting-induced lignification may also be a key contributor to the browning of fresh-cut water-bamboo shoots (45). During 1 week of storage at 20 °C, lignin content in fresh-cut jicama increased from 16.50 to 52.22 mg/kg as browning progressed, and these lignin levels were positively correlated with color changes (46).

These reports indicate that class III peroxidases contribute to tissue browning through two separate mechanisms. While certain peroxidase members directly drive the classic oxidation of phenols into brown polymers (47), other isoforms, such as PtrPO21, specialize in accelerating lignin polymerization (48). This secondary pathway enhances cell wall rigidity, indirectly worsening enzymatic browning. Moreover, specific isoenzymes like longan PRX53–2, which locates in the vacuole and the endoplasmic reticulum, can mediate both phenolic oxidation and lignin polymerization during internal peel browning (49). Therefore, the spatial and substrate-specific distribution of these individual isoenzymes determines the precise mode and severity of discoloration in fresh produce.

Furthermore, it has been reported that lignin synthesis and lignification, mediated by class III peroxidases, are dependent on the presence of H_2_O_2_ (50). During mechanical injury such as cutting, plants intentionally initiate localized ROS burst, generating an abundance of H_2_O_2_ as a crucial signaling molecule and defense response (51). Under these conditions, cell wall-bound anionic peroxidase isoforms such as apoplastic peroxidases with high affinity for monolignols utilize this localized H_2_O_2_ as an essential co-substrate to drive the cross-linking of coniferyl and sinapyl alcohols (52), thereby accelerating lignin biosynthesis.

#### 2.2.3 Manipulating peroxidase activity could regulate enzymatic browning

Numerous studies have demonstrated a strong correlation between peroxidase activity and the browning of fresh-cut fruits and vegetables, with cutting operations known to induce its activity. In fresh-cut taro, both peroxidase activity and the expression of four peroxidase-encoding genes increased as browning progressed during cold storage (4). Similar findings have been reported for other fresh-cut produce, including jicama (46), *Zizania latifolia* (53), Chinese water chestnuts (54), and eggplant fruits (55). Collectively, these reports suggest that fresh-cutting induces peroxidase activity at both gene and protein levels, and the altered activity correlates closely with browning in fresh-cut produce.

Furthermore, many studies have demonstrated that certain methods or technologies can reduce enzymatic browning by regulating peroxidase activity. For instance, in fresh-cut water-bamboo shoots, high-pressure CO_2_ application alleviates lignification and browning by restricting peroxidase activity (45). Similar to this study's context, melatonin treatment reduces phenolic oxidation and ROS metabolism while enhancing peroxidase activity, thereby reducing browning in fresh-cut Chinese water chestnuts (56). Our recent study showed that the application of glycolic acid enhanced peroxidase activity, which subsequently reduced H_2_O_2_ levels and mitigated the browning progression of fresh-cut taro slices during cold storage (4). Therefore, it is proposed that modulating peroxidase activity is a promising strategy for controlling enzymatic browning in fresh-cut produce.

However, many seemingly contradictory results regarding the role of peroxidases in browning have been reported. This divergent outcome suggests that the involvement of peroxidases in enzymatic browning is species-dependent and dictated by the differential regulation of specific isoforms or members under contrasting physiological scenarios. On one hand, certain classical secretory peroxidase isoforms function as pro-oxidants; they directly oxidize phenolic substrates into quinones using H_2_O_2_ as an electron acceptor, thereby accelerating tissue browning. On the other hand, specific peroxidase isoforms or members, particularly those co-regulated with antioxidant systems, primarily act as ROS scavengers. In these anti-browning scenarios, the upregulation of these specific members effectively reduces ROS level, thereby alleviating oxidative stress and indirectly suppressing the overall browning cascade. Therefore, we contend that these inconsistencies arise from a failure to differentiate specific members or isozymes within the peroxidase superfamily.

## 3 Synergistic effects of PPO and peroxidases on browning

PPO is widely recognized as the primary enzyme initiating enzymatic browning in numerous commodities, and it acts synergistically with certain peroxidases to accelerate this process (57, 58), especially class III peroxidases. During PPO-mediated phenolic oxidation, molecular oxygen is reduced to generate H_2_O_2_ (59). This locally produced H_2_O_2_ subsequently serves as a co-substrate that facilitates further phenolic oxidation by specific peroxidases. We therefore propose that PPO amplifies the pro-browning effects of some peroxidase by supplying the H_2_O_2_ required for phenolic oxidation. In addition, PPO and certain peroxidases often act on overlapping phenolic substrates (10, 60, 61), triggering a dynamic interplay of substrate competition and cross-activation that adds another layer of complexity to their combined contribution to enzymatic browning.

## 4 Conclusion and outlook

In this opinion, we emphasize the vital role of peroxidases in the browning process and reviewed their diverse functions, highlighting their integrated capacity to regulate enzymatic browning by orchestrating ROS metabolism, phenolic oxidation, and lignification. Available evidence suggests that peroxidases may exert dual and context-dependent effects on enzymatic browning. The interplay between oxidative stress and secondary metabolism probably dictates the intensity and progression of browning in fresh-cut produce. Therefore, simply labeling peroxidases as “browning promoters” or “ROS scavengers” is both incomplete and misleading. Additionally, given that the specific roles of peroxidase family members vary across superfamilies and different plant species, further research is needed to identify the dominant isoforms within specific crops.
